# Blood Pressure Management Pre- and Post-Reperfusion in Acute Ischemic Stroke: Evidence and Insights from Recent Studies

**DOI:** 10.1007/s11910-025-01443-5

**Published:** 2025-07-25

**Authors:** Feifeng Liu, Craig S. Anderson

**Affiliations:** 1 Department of Neurology, Shanghai East Hospital, School of Medicine, Tongji University, Shanghai, China; 2 The George Institute for Global Health, University of New South Wales, Sydney, Australia; 3 Institute of Science and Technology for Brain-Inspired Intelligence, Fudan University, Shanghai, China; 4 Neurology Department, Royal Prince Alfred Hospital, Sydney, NSW Australia

**Keywords:** Acute ischemic stroke, Hypertension, Blood pressure management, Outcomes, Clinical trials

## Abstract

**Purpose of Review:**

Effective control of blood pressure (BP) is potentially a critical determinant in making a successful recovery from acute ischemic stroke. BP is often elevated after the onset of this critical illness, and it is related to both the severity of the ischemic injury and subsequent changes in cerebral perfusion and collateral blood flow. However, recent studies have challenged longstanding assumptions over the safety and efficacy of BP lowering in being able to reduce the risk of reperfusion injury in acute ischemic stroke. This review synthesizes contemporary evidence and discusses the evolving landscape of BP management in acute ischemic stroke.

**Recent Findings:**

Three prehospital ambulance-initiated trials of early BP lowering in patients with undifferentiated stroke have shown neutral or adverse effects, while the results of multiple trials of in-hospital management BP lowering in patients who have received successful reperfusion therapy for acute ischemic stroke due to large-vessel occlusion highlight the risks of over-aggressive treatment.

**Summary:**

Despite considerable research effort, the optimal BP management strategy in acute ischemic stroke remains uncertain, although avoiding excessive reductions appears critical.

**Supplementary Information:**

The online version contains supplementary material available at 10.1007/s11910-025-01443-5.

## Introduction

Stroke remains the leading cause of death and disability worldwide, for which the global disease burden is likely to further increase in the next 20 years, with ischemic stroke accounting for nearly 70% of all incident stroke [1, 2]. Reperfusion therapy, which includes intravenous thrombolysis (IVT) and/or endovascular thrombectomy (EVT), is now recommended in all guidelines as the key treatment to rapidly restore blood supply to the ischemic brain and improve functional outcome after acute ischemic stroke (AIS) [3–5]. Despite the proven effectiveness of this treatment, only approximately one-third of patients treated with IVT can achieve a good functional outcome [6], and many of the more serious patients fail to achieve independent living despite having successful reperfusion from EVT [7]. These adverse outcomes are related to the complex nature of the evolving ischemic penumbra which is critically dependent on changes of cerebral blood flow and microvascular system [8, 9]. Blood pressure (BP) has a critical influence on both cerebral ischemia and reperfusion injury in AIS, yet the optimal approach to BP management remains uncertain. Although elevated BP is readily modifiable, both excessive hypotension and hypertension lead to poor outcomes [10, 11]. This review synthesizes recent evidence from randomized controlled trials (RCTs) and mechanistic studies on the pathophysiological role of BP in AIS, to provide fresh perspectives on both pre- and post-reperfusion BP management in AIS.

## Pathophysiological Considerations: Cerebral Autoregulation

Cerebral autoregulation exists to maintain brain function through the maintenance of stable cerebral blood flow (CBF) across a range of systemic changes in BP through physiological changes and illness. This has been described as Lassen’s curve, where a wide autoregulatory plateau of mean arterial pressure (MAP) in the range of 60 to 150 mmHg exists under normal conditions [12]. This *static* autoregulation model assumes the existence of symmetric buffering of both increases and decreases in BP.

The directional sensitivity of MAP has key implications in AIS, where cerebral perfusion becomes pressure-passive and vulnerable in the infarct core and ischemic penumbra, respectively [13]. Thus, any associated systemic reduction in BP has the potential to directly diminish CBF and expand the volume of the evolving cerebral infarct, especially in patients with poor collateral status [14, 15]. Poor perfusion pressure may further compromise collateral flow and worsen cerebral ischemia [16]. Conversely, persistent elevation in BP may surpass the upper limit of cerebral autoregulation, causing hyperperfusion, endothelial injury, and hemorrhagic transformation [17]. Large intraprocedural drops in MAP (> 40%) in patients who receive general anesthesia for EVT, and post-procedural elevation in BP, are independently associated with malignant brain edema and symptomatic intracranial hemorrhage (sICH) [18].

Dynamic autoregulation refers to the brain’s ability to maintain stability in response to rapid changes in systemic BP. Unlike the static autoregulation model, which describes the maintenance of CBF across a broad range of BP, dynamic autoregulation emphasizes short-term vascular responsiveness, but which is particularly vulnerable in the context of acute cerebral ischemia [19]. In a recent pooling analysis of over 140 studies in healthy adults, the true plateau of ‘static’ autoregulation appeared to be much narrower (approximately 80–100 mmHg) and for CBF to respond asymmetrically to changes in BP: that is, CBF is more effectively buffered as BP increases than when it decreases [19]. Specifically, in response to decreasing MAP, the slope of CBF is steeper than it is for increasing MAP, suggesting cerebral blood vessels are less effective in protecting the brain from hypoperfusion. These findings imply that below the lower limit of autoregulation, even modest reductions in BP can precipitate a failure to maintaining cerebral perfusion, especially in regions with poor collateral blood flow.

In patients with AIS, especially those with longstanding hypertension, both static and dynamic autoregulatory mechanisms are often impaired [20], producing a pressure-passive state whereby cerebral perfusion becomes directly dependent on systemic BP. In such circumstances, even within *normal* BP parameters, any further reduction in BP can significantly compromise perfusion of the ischemic penumbra. Further support for this mechanism is the clinical observation that blood pressure variability (BPV), not just absolute BP, is an independent predictor of the outcome from AIS. Extreme fluctuations in BP, as well as rapid declines, may further exacerbate tissue hypoperfusion in regions of the brain with impaired autoregulatory capacity. These pathophysiological insights highlight the need for careful, individualized, BP modulation in the early stages of AIS, whereby the goal is to preserve penumbral perfusion without triggering reperfusion injury via stable non-aggressive, rather than abrupt, normalization of BP.

## Blood Pressure Management before Reperfusion Therapy

The optimal BP management in the hyperacute phase of AIS, particularly before a patient receives reperfusion therapy, remains a topic of ongoing debate. Several recent RCTs have examined the effects of BP lowering in the prehospital setting that included patients who were eligible to receive reperfusion therapy. Table 1 summarizes the key design elements of these RCTs. Of these, the fourth Intensive Ambulance-Delivered Blood-Pressure Reduction in Hyperacute Stroke Trial (INTERACT4) was the largest trial on prehospital BP management in patients with suspected stroke conducted across multiple centers in China to determine the effect of ultra-early BP reduction in the ambulance. Patients with a systolic BP ≥ 150 mmHg were randomized either to intensive BP lowering (target systolic BP 130–140 mmHg) or usual care (only treatment of extremely high BP). The trial produced an overall neutral result because of a balance in opposing treatment effects between patients with AIS and intracerebral hemorrhage (ICH): the former had worse functional outcome (common odds ratio [OR] 1.30, 95% confidence interval [CI] 1.06–1.60) whereas there were benefits in those with ICH (OR 0.75, 95%CI 0.60–0.92). The likelihood of these results being due to chance was one in a thousand (p interaction = 0.001) [21].

**Table 1:** Summary of key trials of blood pressure management prior to reperfusion therapy for acute ischemic stroke

Publication year	Trial name	Population / setting	Intervention group	Control group	Primary outcome	Key findings
2019	RIGHT-2 [23]	Suspected acute stroke within 2 h onset, SBP ≥ 150mmHg / prehospital, United Kingdom	Transdermal glyceryl trinitrate for 4 days	Sham patch	90-day mRS (ordinal shift)	No improvement in outcome; trend toward harm in ICH subgroup
2022	MRASAP [22]	Suspected acute stroke within 3 h onset, SBP ≥ 140mmHg / prehospital, Netherlands	Transdermal glyceryl trinitrate for 24 h	Standard care without transdermal glyceryl trinitrate	90-day mRS (ordinal shift)	No functional benefit; safety concerns in ICH patients (early termination)
2024	INTERACT4 [21]	Suspected acute stroke within 4 h onset, SBP ≥ 120mmHg / prehospital, China	Intravenous antihypertensives (mainly urapidil) with a target SBP of 130–140 mmHg within 30 min	Usual care (antihypertensives on ambulance only if SBP ≥ 220mmHg)	90-day mRS (ordinal shift)	No difference overall; worse outcomes in ischemic stroke subgroup
2024	TRUTH [24]	AIS patients with BP > 185/110 mmHg eligible for Intravenous thrombolysis / in hospital, Netherlands	Active BP lowering < 185/110 mmHg using IV antihypertensives	No active BP lowering	90-day mRS (ordinal shift)	No functional benefit; improved IVT access but potential for harm

Note: mRS refers to modified Rankin scale; BP blood pressure; SBP systolic BP; ICH intracranial hemorrhage; AIS acute ischemic stroke; IVT intravenous thrombolysis

The other two prehospital RCTs– the second Prehospital Transdermal Glyceryl Trinitrate in Patients with Ultra-acute Presumed Stroke (RIGHT 2) and Prehospital Transdermal Glyceryl Trinitrate in Patients with Presumed Acute Stroke (MR ASAP) - also failed to demonstrate an overall benefit of early BP control using a transdermal glyceryl trinitrate (GTN), a nitric oxide donor [22, 23]. In MR ASAP, however, there was a trend of the point estimate towards less favorable functional outcome in the glyceryl trinitrate group compared to the no-treatment control group (adjusted common OR 0.67, 95% CI 0.39–1.13) in the 201 patients (62%) with acute ischemic stroke (with or without reperfusion therapy), which was stronger in those patients received intravenous thrombolysis (adjusted common OR 0.51, 95% CI 0.26–0.99).

These findings underscore the complexity of BP management before the use of reperfusion therapy. Whilst permissive hypertension may help to maintain collateral flow to the ischemic penumbra, BP lowering may reduce this below critical thresholds in regions where autoregulation is impaired. This hypothesis is supported by results of the Thrombolysis and Uncontrolled Hypertension (TRUTH) study, a prospective multicenter ‘real world’ observational cluster-based study undertaken in The Netherlands [24]. This study compared two BP management strategies prior to IVT: in 27 hospitals, 853 patients received intravenous antihypertensive treatment to lower BP below 185/110 mmHg before IVT versus in 10 hospitals where 199 patients received IVT only after BP falls spontaneously below this threshold. Despite a shorter door-to-needle time in the active BP-lowering hospitals, there was a trend toward worse functional outcomes for the patients (adjusted OR 1.27, 95% CI 0.96–1.68) and higher rates of sICH (adjusted OR 1.28, 95% CI 0.62–2.62).

A meta-analysis involving over 56,000 IVT-treated AIS patients found that elevated pre-treatment systolic BP was significantly associated with worse functional outcome and increased risk of sICH. Specifically, each 10 mmHg rise in pre-treatment systolic BP increased the odds of sICH by 8% and decreased the likelihood of functional independence (adjusted OR 0.91, 95% CI 0.84–0.98) [25]. Similarly, an individual patient data meta-analysis (*n* = 1753) from the Highly Effective Reperfusion Evaluated in Multiple Endovascular Stroke Trials (HERMES) collaboration found that higher systolic BP (≥ 140 mmHg) at the time patients were admitted to hospital was associated with worse 90-day functional outcome and larger infarct volumes [26]. Of course, such association may not necessarily indicate causality: elevated BP may simply be a risk marker. However, as the totality of the randomized evidence has failed to demonstrate a benefit from early BP lowering therapy before reperfusion but instead has raised concerns over harms if the reduction in BP is large, one should be more cautious over the approach to BP lowering than has been the case over the last decade or more as we have become more comfortable with the use of reperfusion therapies.

Another BP parameter - blood pressure variability (BPV) - is attracting increasingly attention as an important outcome predictor in AIS and other critical illnesses. A post-hoc analysis of the small phase IIb Blood pressure after Endovascular Stroke Therapy (BEST) study, a multi-center prospective cohort multi-arm trial in EVT-treated patients, demonstrated that both increases and decreases in MAP by > 15% before EVT were independently associated with worse 90-day functional outcome. These results further emphasize the importance of hemodynamic stability in the context of reperfusion therapy for AIS [27].

Taken together, all these studies indicate that early intensive BP lowering in patients eligible for reperfusion therapy may be harmful due to compromising autoregulatory capacity of ischemic tissue. The prevailing strategy, therefore, indicates permissive hypertension is acceptable, allowing elevated BP within defined safety thresholds (e.g., < 185/110 mmHg for IVT, < 220/120 mmHg if no IVT is planned) and maintaining modest BPV. The goal of pre-reperfusion BP management is to maintain stability rather than aggressive BP normalization.

## BP Management after Reperfusion Therapy

Whilst there is consistency across studies of an association between elevated BP and increased odds of reperfusion injury, hemorrhagic transformation, and poor outcomes [28, 29], evidence from multiple RCTs in recent years indicates that intensive post-EVT BP lowering is harmful. In particular, the BP-arm of the international Intensive Blood Pressure Reduction with Intravenous Thrombolysis Therapy for Acute Ischemic Stroke Study (ENCHANTED) involving 2196 patients showed that intensive BP lowering (target systolic BP 130–140 mmHg) was not superior to guideline-recommended control (< 180 mmHg) in terms of functional outcome (OR 1.01, 95% CI 0.87–1.17; *p* = 0.87), despite reducing the rate of any ICH (14.8% vs. 18.7%, OR 0.75, 95% CI 0.60–0.94; *p* = 0.0137) [30]. However, this study was undertaken before EVT was widely used as part of standard of care in management of acute stroke and there was only a modest between-group difference in the levels of BP control achieved.

For EVT-treated patients, the follow-up Enhanced Control of Hypertension and Thrombectomy Stroke Study (ENCHANTED2/MT) provided important, if not surprising, results. Through a multicenter RCT comparison of intensive BP lowering to a systolic target < 120 mmHg with a conventional systolic range of 140–180 mmHg in patients following successful EVT for large-vessel occlusive AIS in China, the trial was terminated early after 821 patients were enrolled due to significantly worse clinical outcomes in the intensive group: worse functional recovery (common OR 1.37, 95% CI 1.07–1.76), greater early neurological deterioration (common OR 1.53, 95% CI 1.18–1.97), and increased major disability (common OR 2.07, 95% CI 1.47–2.93) [31]. These findings were unexpected as the earlier, Blood Pressure Target in Acute Stroke to Reduce Hemorrhage After Endovascular Therapy (BP-TARGET) multicenter RCT in France, involving 324 patients to a systolic BP target of 100–129 mmHg versus 130–185 mmHg after successful EVT, showed no significant difference in the primary outcome of any reperfusion ICH (42% vs. 43%, adjusted OR 0.96, 95% CI 0.60–1.51) nor of favorable functional outcome (44% vs. 45%, adjusted OR 0.93, 95% CI 0.58–1.48), despite more episodes of extreme hypotension in the intensive compared with control groups (8% vs. 3%, adjusted OR 2.53, 95% CI 0.86–7.41) [32]. Subsequently, the Outcome in Patients Treated with Intra-Arterial Thrombectomy–Optimal Blood Pressure Control (OPTIMAL-BP) trial conducted in Korea showed that functional independence was significantly lower in the intensive group (systolic BP < 140 mmHg) compared to the control group (140–180 mmHg) in patients who had had successful EVT (39.4% vs. 54.4%, adjusted OR 0.56, 95% CI 0.33–0.96; *p* = 0.03) [33]. The Blood Pressure After Endovascular Stroke Therapy-II (BEST-II) study, which tested three post-EVT systolic BP targets (< 140, < 160, and ≤ 180 mmHg), showed no improvement in infarct volume or 90-day functional outcome in the lower BP groups, although it was not designed for the testing of superiority. However, the probability of a benefit being realized from the control of systolic BP to lower targets was judged to be too low to be feasibly tested in a subsequent phase 3 RCT [34]. A pooled analysis of these four RCTs concluded that intensive post-EVT control of BP is significantly likely to reduce the likelihood of functional independence (OR 0.68, 95% CI 0.5–0.91) without reducing the odds of sICH (OR 1.14, 95% CI 0.77–1.69) or death (OR 1.16, 95% CI 0.87–1.55) [35]. A summary of these key RCTs is presented in Table 2.

**Table 2:** Summary of key trials of blood pressure management after reperfusion in acute ischemic stroke

Publication year	Trial name	Population / setting	Intervention group	Control group	Primary outcome	Key findings
2019	ENCHANTED (BP Arm) [30]	AIS patients treated with intravenous alteplase, SBP ≥ 150 mmHg / international	SBP 130–140 mmHg for 72 h	Guideline-recommended SBP < 80 mmHg	90-day mRS (ordinal shift)	No improvement in functional outcome, but reduced ICH
2021	BP-TARGET [32]	AIS patients post-EVT with successful reperfusion / France	SBP 100–129 mmHg for 24 h	SBP 130–185 mmHg	Radiographic ICH at 24–36 h	No difference in ICH; slightly more hypotension in intensive group
2022	ENCHANTED2/MT [31]	AIS post-EVT with successful reperfusion; SBP ≥ 140 mmHg / China	SBP < 120 mmHg for 72 h	SBP 140–180 mmHg	90-day mRS (ordinal shift)	Worse functional outcomes with intensive BP control; (early termination)
2023	OPTIMAL-BP [33]	AIS post-EVT with SBP ≥ 140 mmHg / Korea	SBP < 140 mmHg for 24 h	SBP 140–180 mmHg	90-day functional independence (mRS 0–2)	Lower rate of functional independence in intensive group; no difference in ICH or mortality
2023	BEST-II [34]	AIS post-EVT with successful reperfusion / USA	SBP < 140 mmHg or SBP < 160 mmHg for 24 h	SBP ≤ 180 mmHg	Infarct volume and 90-day utility-weighted	No clear benefit from lower targets; low probability of benefit in larger trial

Note: mRS refers to modified Rankin Scale; BP blood pressure; SBP systolic BP; ICH intracranial hemorrhage; AIS acute ischemic stroke; EVT endovascular thrombectomy

An observational study suggests that the spontaneous decline in BP that follows successful EVT is a physiological marker of reperfusion and favorable autoregulatory response [36]. Moreover, in a study of Dias et al. involving 458 AIS patients who underwent EVT, a spontaneous decline in SBP within the first hour post-EVT was independently associated with early dramatic neurological recovery (OR 1.14 per 10 mmHg drop; *p* = 0.04), albeit without any significant association with improved 90-day functional outcome [37]. These findings support the hypothesis that the normalization of BP reflects natural cerebral autoregulation with reperfusion rather than being a therapeutic effect of BP lowering intervention.

Collectively, these results challenge the prevailing assumption that for BP, ”lower is better after recanalization” in the post-reperfusion period [38]. The lack of benefit, and in some cases, evidence of harm, from intensive BP reduction suggests that elevated BP may be physiologically adaptive in the context of impaired cerebral autoregulation. Thus, overly aggressive BP lowering risks compromising ‘at-risk’ cerebral perfusion.

## Special Considerations: Hypotension, Induced Hypertension, and BPV

Hypotension during AIS, whether spontaneous or iatrogenic, has consistently been associated with larger infarct volumes and worse clinical outcomes, particularly in patients undergoing general anesthesia for EVT [39–42]. Thus, induced hypertension has emerged as a potential strategy to augment collateral perfusion. In a small pilot trial, the use of phenylephrine-induced hypertension (target increases in SBP of 20% without exceeding 200 mmHg) was found to be feasible, safe, and associated with neurological improvement in selected AIS patients [43]. Similarly, the Safety and Efficacy of Therapeutic Induced Hypertension in Acute Non-Cardioembolic Ischemic Stroke (SETIN-HYPERTENSION) trial showed a benefit of induced hypertension in patients with progressive stroke [44], while there was no significant difference in 90-day functional outcome across three randomized groups of different systolic BP targets in non-IVT treated AIS patients (140–160 mmHg, 161–180 mmHg, and 181–200 mmHg) within 12 h of symptom onset in the Early Manipulation of Arterial Blood Pressure in Acute Ischemic Stroke (MAPAS) trial [45]. However, MAPAS showed several other aspects: sICH was more frequent in the highest SBP target group (9% vs. 1–3%; *p* = 0.049), which also had greater drug-related adverse events of acute coronary syndromes and bradyarrhythmia; a logistic regression analysis showed the most favorable outcome for patients allocated to the mid-range group (SBP 161–180 mmHg, OR 2.82; *p* = 0.03) compared to the highest group; and patients with clinical-radiological mismatch had significantly worse outcomes when their BP was reduced compared to those in whom the BP was either increased or unchanged. Again, these data highlight the importance of individualized BP management based on imaging or clinical markers of salvageable tissue.

Another pilot RCT, uniquely focused on intra-procedural BP augmentation during EVT under general anesthesia, showed the feasibility and safety of achieving an SBP target of 160–180 mmHg during EVT [46]. However, the small sample size (*n* = 52) precluded any definitive conclusions to be drawn regarding the impact on early neurological recovery or longer term functional outcome. A large multicenter phase III trial is currently in progress to assess the efficacy of intraoperative augmentation of systolic BP (target 170 ± 10 mmHg vs. 140 ± 10 mmHg) during EVT under general anesthesia [47]. Thus, induced hypertension should be considered investigational at this time and not part of rontine AIS care. It should be tested within RCTs or used cautiously in highly selected patients with salvageable tissue identified by imaging, where individualized BP augmentation may offer potential benefit [48].

BPV is receiving considerable attention as a focus for hemodynamic investigation in AIS. There is increasing evidence to suggest that BPV, across all the various indices of standard deviation, coefficient of variation, and average real variability, is independently associated with the poor clinical outcomes of increased infarct growth, hemorrhagic transformation, death and physical function across all phases of stroke [49–51]. However, therapeutic strategies to minimize BPV are yet to be defined. Expert opinion and observational data favor the use of long-acting antihypertensive agents, to avoid abrupt falls in BP, and with the use of protocols that encourage the smooth titration of BP [52]. Moreover, it has been reported that during EVT, BPV is greater with general anesthesia than concious sedation [53], suggesting that anesthetic choice can influence hemodynamic stability. The dynamic instability in cerebral autoregulation provides a mechanistic rationale for the association between BPV and poor outcome [19]. The emerging paradigm is that post-ichemia BP management should be prioritized not simply by reaching of a numerical systolic target, but for a gradual and steady reduction aligned with impaired cerebral hemodynamic adaptability. Of course, the causal relationship between BPV and outcomes in AIS remains to be fully validated but BPV, like an elevated BP alone, may simply reflect the severity of the underlying disease rather than being directly contributing to adverse outcomes.

## Blood Pressure Agent Selection

Current guideline recommend various intravenous antihypertensive agents that allow a continuous infusion and ability to titrate BP control, such as labetalol, nicardipine, or clevidipine, to achieve target BP thresholds during peri-perfusion therapy for AIS, whereas other agents such as hydralazine, enalaprilat, or nitroprusside are recommended to be reserved to as initial boluses and certain situations [3]. However, these recommendations are primarily based on expert opinion, on the basis of the drugs having a rapid onset, ease of titration, and favorable safety profile in the acute stroke setting. Importantly, though, there are no RCTs that have directly compared different classes of antihypertensive agents during the perireperfusion period and to evaluate their impact on clinical outcome. Existing RCTs have mainly focused on the timing and intensity of BP reduction rather than for headtohead drug comparisons, which is understandably more complex. For example, the INTERACT4 and ENCHANTED 2/MT studies, primarily used intravenous urapidil (a selective peripheral α₁adrenergic antagonist with central 5hydroxytryptamine 1 A receptor agonist activity) [21, 31], while the MR-ASAP and RIGHT2 trials investigated transdermal glyceryl trinitrate [22, 23]. The BP-TARGET, BEST-II, OPTIMAL-BP, and TRUTH studies mainly applied nicardipine for acute BP management [24, 32–34]. The ENCHANTED BP arm utilized regionally available agents, such as nitroglycerin, labetalol, urapidil, sodium nitroprusside, and nicardipine [30]. A retrospective study of 784 AIS patients in the United States suggested that intravenous clevidipine, nicardipine, and nitroprusside all effectively lower systolic blood pressure without increasing SBP variability [54]. A post-hoc regional analysis of the ENCHANTED trial revealed marked differences in early BP management: urapidil and sodium nitroprusside were mainly used in China, nicardipine in other Asian countries, labetalol and nitroglycerin in Western countries, and sodium nitroprusside in South America [55]. The choice of antihypertensive agents varies across regions, likely due to differences in local clinical practice patterns, drug availability, and physician preferences.

As the usefulness of druginduced hypertension remains uncertain and largely investigational, some studies have used phenylephrine, other vasoactive agents, or fluids to achieve target BP [43–45]. EVT studies have also attempted anesthesiologistled BP control using vasoconstrictors such as metaraminol, with anesthetic adjustments and fluid therapy [46, 47].

## Future Directions and Challenges

Despite the achievements of several RCTs to date, the optimal management of BP in AIS remains elusive and likely to vary depending on patient-specific factors, such as collateral status, autoregulation capability, and baseline BP. Current recommendations favor maintaining a BP < 185/110 mmHg or 180/105 mmHg before and for the first 24 h after IVT, respectively [3, 5], but these thresholds are not individualized. A recent pre-specified secondary analysis of ENCHANTED2/MT indicates that the adverse effect of intensive BP lowering is strongly influenced by the degree of collateral status, which suggests that BP management based on imaging stratification might be worthwhile [56]. Moreover, it has been reported that any deviation in BP beyond the personalized limits of cerebral autoregulation after successful EVT is associated with worse clinical outcomes, including infarct growth and hemorrhagic complications, which supports the concept of autoregulation-guided individualized BP management in AIS [57, 58].

Individualizing BP management strategies is a promising direction for future research [59]. Ongoing RCTs such as the Management of Systolic blood pressure during Thrombectomy by Endovascular Route for acute ischaemic stroke (MASTERSTROKE) [47], the multi-factorial, multi-arm, multi-stage, randomised, global adaptive platform trial for stroke (ACT-GLOBAL) [60], and the second Outcome in Patients Treated with Intra-Arterial Thrombectomy-Optimal Blood Pressure Control (OPTIMAL-BP 2) [61] are all expected to shed further light on this topic. The MASTERSTROKE trial aims to determine whether targeting a higher SBP (170 ± 10 mmHg) during general anesthesia for EVT improves 90-day functional outcomes in patients with acute anterior circulation AIS, compared to the standard target of 140 ± 10 mmHg [47]. ACT-GLOBAL is evaluating three BP lowering strategies in AIS patients with elevated sytolic BP after EVT: a conservative approach whereby BP is reduced by 5–10 mmHg if necessary or to a target of 175–180 mmHg if baseline SBP ≥ 180 mmHg; a moderate approach whereby BP is reduced by 10–20 mmHg or to a target of 160 ± 5 mmHg (whichever is higher); and an intensive approach (SBP reduction by 30–50 mmHg or a target of 140 ± 5 mmHg (whichever is higher) [60]. OPTIMAL-BP-2 aims to evaluate whether targeted SBP elevation (a 20% increase from baseline, capped at 180 mmHg) improves outcomes in patients with SBP < 140 mmHg after successful EVT [61]. These studies highlight the growing interest in a tailored rather than uniform approach to BP loweing to further advance the paradigm of individualized hemodynamic management in AIS patients.

## Conclusions

BP is clearly a potentially readily modifiable factor to improve the outcome from AIS, but the optimal approach before and after reperfusion therapy remains uncertain and increasingly complex. The current randomized evidence indicates that early and intensive BP lowering before reperfusion is harmful in AIS due to the ability to compromise cerebral autoregulation. Thus, there is no strong evidence to support a shift in the longstanding guideline recommendation of aiming for a systolic BP < 180 mmHg after IVT. However, one should adopt a cautious approach to such treatment to avoid extreme reductions and variability in BP. Future strategies should emphasize individualized, physiology-informed, BP management to improve the outcome for patients with AIS.

## Supplementary Information

Below is the link to the electronic supplementary material.

Supplementary Material 1

Supplementary Material 2

## Data Availability

No datasets were generated or analysed during the current study.

## Key References

- Wang Y, Payne SJ. Static autoregulation in humans. J Cereb Blood Flow Metab. 2024;44:1191–1207. 10.1177/0271678X231210430.
  – Systematic review of 143 studies in healthy adults reveals that cerebral autoregulation is more effective at buffering increases in arterial blood pressure than decreases, with a narrower autoregulatory plateau (around 80–100 mmHg) than traditionally believed.
- Li G, Lin Y, Yang J, Anderson CS, Chen C, Liu F, et al. Intensive ambulance-delivered blood-pressure reduction in hyperacute stroke. N Engl J Med 2024;390:1862–1872. 10.1056/NEJMoa2314741.
  – Large, China-based RCT of 2404 suspected stroke patients comparing prehospital intensive BP lowering (SBP target 130–140 mmHg) vs. usual care. No overall benefit in functional outcomes, but subgroup analysis showed improved outcomes in hemorrhagic stroke and worse outcomes in ischemic stroke.
- Zonneveld TP, Vermeer SE, van Zwet EW, Groot AED, Algra A, Aerden LAM, et al. Safety and efficacy of active blood-pressure reduction to the recommended thresholds for intravenous thrombolysis in patients with acute ischaemic stroke in the netherlands (truth): A prospective, observational, cluster-based, parallel-group study. Lancet Neurology 2024;23:807–815.10.1016/S1474-4422(24)00177-7.
  – Prospective, observational, cluster-based study across 37 Netherlands stroke centres comparing active blood-pressure-lowering versus non-lowering strategies in acute ischaemic stroke patients with BP > 185/110 mm Hg eligible for intravenous thrombolysis. Active lowering increased thrombolysis rates and reduced door-to-needle times but did not significantly improve 90-day functional outcomes.
- Yang P, Song L, Zhang Y, Zhang X, Chen X, Li Y, et al. Intensive blood pressure control after endovascular thrombectomy for acute ischaemic stroke (ENCHANTED2/MT): a multicentre, open-label, blinded-endpoint, randomised controlled trial. Lancet 2022;400:1585–1596. 10.1016/S0140-6736(22)01882-7.
  – Multinational RCT enrolling 1000 patients with acute ICH to compare intensive BP lowering (SBP < 120 mmHg) with guideline-recommended target (SBP 140–180 mmHg). No benefit on functional outcomes; intensive BP lowering was associated with a non-significant trend toward harm and more renal adverse events.
- Palaiodimou L, Joundi RA, Katsanos AH, Ahmed N, Kim JT, Goyal N, et al. Association between blood pressure variability and outcomes after endovascular thrombectomy for acute ischemic stroke: an individual patient data meta-analysis. Eur Stroke J 2024;9:88–96. 10.1177/23969873231211157.
  – Individual patient data meta-analysis showing that higher 24-hour blood pressure variability after endovascular thrombectomy for acute ischemic stroke is independently associated with increased risk of death and disability.
